# Cultural adaptation to Brazil of the questionnaire Costs of caring for children with cancer^1^

**DOI:** 10.1590/0104-1169.3077.2456

**Published:** 2014

**Authors:** Raquel Pan, Amanda Rossi Marques, Bruna Domingos dos Santos, Eufemia Jacob, Claudia Benedita dos Santos, Lucila Castanheira Nascimento

**Affiliations:** 2 Doctoral student, Escola de Enfermagem de Ribeirão Preto, Universidade de São Paulo, WHO Collaborating Centre for Nursing Research Development, Ribeirão Preto, SP, Brazil; 3 Master's student, Escola de Enfermagem de Ribeirão Preto, Universidade de São Paulo, WHO Collaborating Centre for Nursing Research Development, Ribeirão Preto, SP, Brazil; 4 Undegraduate student in Nursing, Escola de Enfermagem de Ribeirão Preto, Universidade de São Paulo, WHO Collaborating Centre for Nursing Research Development, Ribeirão Preto, SP, Brazil. Scholarship holder from Conselho Nacional de Desenvolvimento Científico e Tecnológico (CNPq), Brazil; 5 PhD, Assistant Professor, School of Nursing, University of California, Los Angeles, California, Los Angeles, USA; 6 PhD, Associate Professor, Escola de Enfermagem de Ribeirão Preto, Universidade de São Paulo, WHO Collaborating Centre for Nursing Research Development, Ribeirão Preto, SP, Brazil

**Keywords:** Neoplasms, Child, Adolescent, Family, Validation Studies, Oncologic Nursing

## Abstract

**OBJECTIVE:**

to present the cultural adaptation of the questionnaire Costs of caring for children with cancer, offering a valid and reliable tool to assess the economic repercussions of childhood cancer for Brazilian families.

**METHOD:**

it is a methodological research with a cross-sectional design. The methodological framework to validate the questionnaire was a combined process that included seven steps: translation to Portuguese; first translated consensus version; evaluation by Expert Committee; consensus on the Expert Committee version; back-translation; consensus of back-translated versions; semantic validation. The study was conducted in two phases: phase one was the translation and back-translations process, with five expert committee members. Phase two was the semantic validation, with 24 participants, who answered an instrument about their impressions of the questionnaire and suggested modifications.

**RESULTS:**

in phase one, items were included, excluded, and replaced to make the content equivalent and valid for use with Brazilian context. In phase two, the majority of the participants were mothers, who made suggestions about the relevance and clarity of the items in the questionnaire.

**CONCLUSIONS:**

the authors discussed these recommendations and made adaptations, turning the questionnaire into a valid and reliable tool for application.

## Introduction

Childhood cancer exerts great financial impact on children and their families. Costs for families may be direct, indirect and psychosocial. Direct costs are related to actual finances associated with treatment, rehabilitation and general care expenses. Indirect costs may include a loss of professional productivity, remunerated and non-remunerated work, such as cleaning and domestic responsibilities. Psychosocial costs are decreasing psychological and social conditions in children and other family members. These direct, indirect, and psychosocial repercussions may contribute to the loss of daily routines of these families. Treatment related hospitalizations or health related care at home make it challenging to maintain their earlier responsibilities, while at the same time coping with the health care demands imposed by the cancer diagnoses and treatments^(^ 1 ^)^. The parents' role as caregivers of sick children changes with the new responsibilities that affect the time they have available, often implying in the loss of their employment^(^ 2 ^)^. Psychological adjustments are needed^(^ 2 ^)^ and, when they have other children, parental responsibilities need to be reorganized^(^ 3 ^)^.

Most of the caregivers who stay during hospitalization with their child with cancer are female, specifically mothers^(^ 2 ^)^. These women had lower education levels, often become unemployed, and have insufficient financial resources^(^ 4 ^)^. The focus of their lives is the care of their sick children, most often totally directing their attention at the sick child, even if they have other children at home. This new situation may cause conflicts in the family with other children and the husband/partner^(^ 4 ^)^. In some families, one or both parents must quit a job to dedicate time to their child's treatment^(^ 5 ^)^. Consequently, family income from these women or from the couple was compromised.

Most families of children with cancer often have limited time available due to the health care demands of children. Parents may lose their job, at a critical time when they need financial resources^(^ 6 ^-^ 7 ^)^ to cover the high costs of medications and medical care. Other financial expenses were incurred with transportation and meal expenses during hospitalizations and medical office visits^(^ 7 ^)^.

Changes in family dynamics, marital relationships, and social support entail additional difficulties that contribute to the increase in family costs. Families often restricted leisure activities or incurred additional debts to maintain normal activities. Families may increase loans through credit cards, or they may sell family goods or properties. Some parents held a second job or organized funding events to meet the financial burdens associated with health care of their children with cancer^(^ 7 ^)^.

Other negative consequences include changes in the parents' professional life. A study^(^ 8 ^)^ developed in Sweden have associated the diagnosis of childhood cancer with both short- and long-term impact on parent's work status. Comparing the time of the diagnosis with one year after the end of the treatment, fewer fathers were at work and a greater proportion of mothers were on sick leave. Although the economic and working situation could be normalized with time, the income of the family was affected negatively after the diagnosis until three months after the end of the treatment^(^ 8 ^)^.

Given all the direct, indirect, and psychosocial costs associated with having children with cancer, there is very little in the literature that measured or quantified these costs, particularly in the context of Brazilian families. However, there is a questionnaire available in English called the *Costs of caring for children with cancer* ^(^ 9 ^)^. This questionnaire has not been translated in Portuguese, and has not been tested for use in Brazilian families. Therefore, the purpose of this study was to present the cultural adaptation of this questionnaire, offering a valid and reliable tool to evaluate the economic repercussions of childhood-juvenile cancer in Brazilian families.

## Method

### Study design

In this methodological research with a cross-sectional design, a questionnaire to evaluate the family costs of care delivery to children and adolescents with cancer was subject to cultural validation.

The methodological framework to validate the questionnaire was an adaptation of the DISABKIDS Group's steps^(^ 10 ^)^. Another step indicated by Guillemin^(^ 11 ^)^, assessment by an Expert Committee, was added to the Group's steps. The combined process included seven steps: 1. Translation to Portuguese (initial translation); 2. First translated consensus version (translation I); 3. Evaluation by Expert Committee; 4. Consensus on the Expert Committee version (translation II); 5. Back-translation; 6. Consensus of back-translated versions (translation III); 7. Semantic validation of items.

The study was implemented between March 2010 to December 2012, and it was conducted in two phases: 1) translation and back-translation of the questionnaire *Costs of caring for children with cancer* ^(^ 9 ^)^, which included these six steps explained before; and 2) semantic validation of the questionnaire adapted to the Brazilian Portuguese language and to the Brazilian culture.

### Ethical aspects

The authors^(^ 9 ^)^ of the original questionnaire were contacted to obtain permission to adapt and translate the instrument for use in Brazilian families. Approvals were received from the Institutional Review Board at the University of São Paulo at Ribeirão Preto College of Nursing (Process 1256/2010) and the National Research Ethics Committee (CONEP; Process 192/2011). Approvals were also received from the directors of the pediatric clinic and the Pediatrics and Child Care Department of the University of São Paulo at Ribeirão Preto Medical School *Hospital das Clínicas *(HCFMRP/USP).

Consent to participate in the research was obtained, in compliance with the guidelines of National Health Council Resolution 196/96, using Informed Consent Forms (ICF). Details about the research were provided to participants, including research objectives, data collection procedures, and the importance of using the ICF copy, contact information of the researchers in case of any questions or concerns, possible risks and benefits, and freedom to drop out of the research at any time. Participants were informed that all responses were anonymous and confidential. Two copies of the ICF were signed.

### The Questionnaire

The questionnaire *Costs of caring for children with cancer *was originally developed in English by authors from the United Kingdom^(^ 9 ^)^. This questionnaire has 58 questions that included items: 1) demographic characteristics of parents (education, marital status, ethnic origin, number of children in the family, age children, gender); 2) clinical information (diagnosis, time since diagnosis, current treatment status); 3) current expenses related to illness (transportation to the hospital, clothing, phone calls); 4) employment (full/part time status at the time of the diagnosis, as well as after diagnosis, employer attitude, role change, hours worked since the diagnosis, absence from work), and 5) public or charitable financial support received (sources, medical benefits, difficulties related to the application process for these benefits).

### Phase I - Translation and Back-Translation

The translation was the first phase in the adaptation process of the questionnaire *Costs of caring for children with cancer* ^(^ 9 ^)^. Two authors fluent in English and familiar with the research theme independently translated the questionnaire from the original English language to Brazilian Portuguese (translation I). Secondly, an assessment process was added to enhance the scientific rigor of the process. The assessment was made by an Expert Committee that included five nurses who were knowledgeable and experienced in the theme. The authors discussed and developed a consensus version.

The Expert Committee used an instrument for assessing equivalence between the original and translated versions of the questionnaire. This instrument was composed by multiple choice questions, which assessed whether the item was translated in accordance with the following assumptions: a) conceptual equivalence; b) equivalence of items; c) semantic equivalence; d) operational equivalence; and e) content validity^(^ 12 ^-^ 14 ^)^. This instrument was designed to facilitate the analysis of the translation process developed by experts.

The Expert Committee separately assessed all questionnaire items, according to the properties included in the evaluation instrument. The Expert Committee was instructed to mark the items with an X in the corresponding space, according to the following criteria: -1= not equivalent (does not agree with the translation); 0= inconclusive; +1= equivalent (agrees with the translation). In case of -1 or 0, the Expert Committee was instructed to include comments and suggestions on the lines below each instrument item.

The research team analyzed both versions, conciliated the translations and drafted a final translated version (translation II). Two bilingual persons who were unfamiliar with the main theme back-translated the questionnaire back to English. The back-translated questionnaire was then forwarded to the original authors in order to analyze and deliberate on the changes that were needed during the cultural adaptation process. The meanings and objectives of the final translated version were the same as the English version, resulting in an adapted Portuguese version for use in Brazilian families.

### Phase II - Semantic Validation

Three authors developed in the semantic validation phase. Parents or caregivers of children with cancer were interviewed about their impressions on the questionnaire. An interview instrument from the Group^(^ 10 ^)^ was used for semantic validation. The interview instrument consisted of three multiple-choice and two open questions. The multiple-choice questions assessed: 1) whether the items and questions in the translated questionnaire were relevant to the situation they experience; 2) if they faced any difficulties with understanding the items in the questionnaire, and 3) if the items were clear and consistent. The open questions assessed: 1) how the participants understood the meaning of the question, and 2) if they found it necessary, how they would reformulate the item or question in their own words.

To facilitate participants' evaluation, the Group^(^ 10 ^)^ divided the questionnaire into subgroups, with a view to guaranteeing more reliable answers. The questionnaire had ten sections (sections A-J). The research team decided to make groups of two or three sections, ranging from 12 to 17 questions. These numbers of questions were thought to be reasonable so that each participant could evaluate the items appropriately, and minimize fatigue, time and effort for each participant. Each subset of items was then evaluated by three parents or caregivers for the children with cancer.

### Participants and data collection site

### Phase I - Translation and Back-Translation

Five experts in pediatric and oncology nursing who were fluent in English were invited to be part of the Expert Committee. The authors developed this phase through face to face meetings at the University of São Paulo at Ribeirão Preto College of Nursing, and electric mail communications.

### Phase II - Semantic Validation Phase

Parents or caregivers of 24 children and adolescents with cancer who were being followed at the HCFMRP/USP were invited to participate. They had to be the family member actively involved in the care of the sick child, such as mothers, fathers, siblings over 18 years of age, maternal or paternal grandparents, who could provide essential information to understanding the economic repercussions of cancer in the family. Their children had to be: 1) under the age of 16 at the time of the cancer diagnosis; 2) younger than 20 years at the time of parent participation in the study; 3) followed at the pediatric clinic or infant outpatient clinic of the research institution; and 4) living with their parents. In the event that both parents were present, only one was asked to participate. Children and adolescents suffering from other chronic conditions associated with cancer, like Down syndrome for example, were excluded, as well as parents and/or responsible caregivers without minimal literacy skills to understand the questionnaire items, as verified by health professionals at the clinic who were more familiar with them. The interviews were done at the Pediatric Inpatient Unit and Pediatric and Child Care Outpatient Clinic of HCFMRP/USP, where children and adolescents received treatment and follow-up care.

### Data analysis

### Phase I - Translation-Back Translation

Descriptive statistics were used to analyze the agreement of Expert Committee members on items on the questionnaire. The database was structured in an EXCEL worksheet (Microsoft Office, 2010) to code the variables. The double-entry technique was used to minimize possible data processing errors. Then, the data were exported to Statistical Package for the Social Sciences (SPSS) software, version 16.0, to calculate frequencies and percentages. Items with agreement levels of 80% or more among the participants were considered^(^ 15 ^)^.

### Phase II - Semantic Validation

Descriptive statistics were used to determine frequencies and percentages of agreement on the items on the multiple choice questions. Items with agreement levels of 80% or more among the parent participants were considered^(^ 15 ^)^. The same analysis process was applied as in the Expert Committee phase.

For open ended responses, related to reformulation and wording of items on the questionnaire, the suggestions were analyzed through discussions among the authors about the relevance of the suggested reformulations. Items that most closely approximated the Brazilian context were considered for adaptation of the questionnaire.

## Results

### Phase I - Translation-Back Translation

The translation process to Brazilian Portuguese took three months. To achieve a cultural adaptation that reflected the Brazilian context, extensive literature searches and discussion meetings with experts were made. Discussions were related to the rights of cancer patients, and therefore, included discussions with one faculty member with expertise in patient law, one lawyer and one social worker. The discussions with the social worker included issues related to available benefits and family leaves allowed by Brazilian legislation for patients and parents of children with chronic illnesses (not specific to cancer). These issues also emerged from her professional experience in support groups for parents of children with cancer.

The translated questionnaire was forwarded to the Expert Committee for evaluation. After all Committee members had evaluated the questionnaire, a first version was obtained. Most items had 80% or more agreement among the Expert Committee^(^ 15 ^)^. Consensus could not be reached on some items, such as those related to education levels, and ethnicity categories, which was adapted by using skin color classification appropriate to the Brazilian context. The Expert Committee raised questions about these items, requiring that adaptations be made and resubmitted to this Committee. To adequate the questions without consensus, the Expert Committee provided citations from the literature. The questionnaire was then back-translated to English. The back-translated English version was forwarded to the original authors for evaluation, who approved the Brazilian Portuguese version of the questionnaire.

For cultural adaptation to Brazilian population, six changes were made. First, was the discrepancy between the geographic dimensions in Brazil and the United Kingdom, conversion was made from miles to kilometers. Second, questions about benefits and aids for patients and their parents needed to be changed completely, because these were different from the United Kingdom. Brazil did not have legal support to protect parents of children and adolescents with cancer, and few benefits were available to patients. Third, content about the parents or caregivers' type of work and leave of absence were changed according to Brazilian legislation. For example, self-employed work and leave of absence need to be clarified. Fourth, the word "helpful" was commonly translated as *útil* in the Brazilian culture. This word was replaced with *ajuda*, in the belief that *útil* would sound pejorative. Fifth, questions about the participants' ethnic origin were changed in accordance with the Brazilian Institute for Geography and Statistics' classification (IBGE)^(^ 16 ^)^. The distinctions among yellow, white, indigenous, mulatto and black people were made due to the Brazilian historical miscegenation and, finally, the question about education was changed completely. The original questionnaire, education involved the age when the individual stopped studying. In Brazil, there were wide variations in the age when education stopped, depending on the financial and social conditions and values. Therefore, we adopted the division according to education level defined in Brazilian law that regulates education, Law 9.394, issued in 1996^(^ 17 ^)^.

### Phase II - Semantic Validation

The semantic validation phase was spread over five months; two months for data collection and three months for data analysis. Participants were 20 mothers, three grandmothers and one sister. The level of education for half of the participants was primary education and the other half was secondary education, which is consistent with the recommendation by the Group^(^ 10 ^)^, to increase representativeness of the sample.

The instrument used for the interviews allowed the participants to make changes, so that the items are clearer on the questionnaire. There were 24 reformulations that were suggested. The research team analyzed the significance, keeping in mind the Brazilian population and the objectives of the translated and culturally adapted questionnaire. Five reformulations were incorporated in the Brazilian Portuguese version. These included items related to: 1) how frequently the child and the responsible caregiver attend the treatment center; 2) what they know about the social rights of cancer patients; 3) receiving a social benefit *Benefício de Prestação Continuada* (BPC) and about the result of the BPC application; 4) how long the parents or caregivers were absent from work; and 5) color classifications. These changes were made to increase the interviewee's understanding of the questions/items and appropriateness of the questionnaire in Brazilian families.

## Discussion

The study demonstrated the cultural adaptation of the Brazilian version of the questionnaire, *Costs of caring for children with cancer*, originally developed for the British population. The instrument was used for evaluating the economic repercussions of children's cancer in British families. We followed a combined process of the two approaches^(^ 10 ^-^ 11 ^)^ to translate and culturally adapt this questionnaire for use in Brazilian Portuguese speaking families. We made modifications that included and excluded items^(^ 15 ^)^.

To our knowledge, this is the first Brazilian Portuguese questionnaire that specifically evaluated the family costs of care in children with cancer. Because of the lack of research devoted to this topic, we reviewed the literature and adapted the tool developed by the original authors^(^ 9 ^)^, and found that their questionnaire *Costs of caring for children with cancer* has the potential to make important contributions to pediatric nursing in Brazilian families of children and adolescents with cancer.

Brazil is a country of continental dimensions and different cultural and social contexts, which can affect the adaptation and validation process about instruments for use in Brazilian parents or caregivers^(^ 10 ^)^. It is, therefore, important to note that this was not a multicenter research, and would not be feasible to implement in other Brazilian regions, without further semantic validation in other areas. We found that the combined methods were useful and increased our understanding of the applicability of the questionnaire in Brazilian families in our area.

We recommend the application of our adapted Brazilian Portuguese version of the *Costs of caring for children with cancer* in future research, which is in progress at the time of this writing. Our preliminary findings have the potential to increase our understanding of the financial costs in families of children and adolescents with cancer, in a specific population followed at the HCFMRP/USP.

It is important to note that the cultural adaptation of the questionnaire was intended for research purposes, and not for routine clinical use of this questionnaire. However, the questionnaire may be useful for characterizing the costs of families attending different oncology treatment centers with the future intent of developing public laws and policies that meet the socioeconomic and health needs of families of children and adolescents with cancer.

## Conclusions

We culturally adapted and validated the *Costs of caring for children with cancer* questionnaire for use in Brazilian Portuguese families, using the combined rigorous methods.

We found that parents, grandparents, and sibling participants in our study understood the items on the questionnaire and it was satisfactorily and acceptable. They had no difficulty with the items during the entire cultural adaptation process, and the Brazilian Portuguese version reflected the Brazilian popular vocabulary and culture. The recommendations by the parents, grandparents and sibling participants were incorporated during the semantic validation phase, which permitted to further improve the adaptation of the questionnaire to the Brazilian language and culture.

The Brazilian Portuguese version of the questionnaire is now available, which represents an important contribution to family nursing research. The questionnaire could facilitate studies related to the economic repercussions and advance the research related to psychosocial care delivered to families of children with cancer. The available questionnaire could also allow professionals to assess the financial repercussions of cancer within the family, and enabling them to assist the family members to mobilize internal and external resources to help them cope with the disease, and consequently lead to comprehensive health promotion for patients and their families.

## References

[1] Aung L, Saw SM, Chan MY, Khaing T, Quah TC, Verkooijen HM (2012). The Hidden Impact of Childhood Cancer on the Family: A
Multi-Institutional Study from Singapore. Ann Acad Med Singapore..

[2] Beck ARM, Lopes MHBM (2007). Cuidadores de crianças com câncer: aspectos da vida
afetados pela atividade de cuidador. Rev. bras. enferm..

[3] Barbeiro FMS (2013). Sentimentos evidenciados pelos pais e familiares frente
ao diagnóstico de câncer na criança. Rev Pesqui: Cuidado é Fundamental Online [Internet]..

[4] Vinhal LM, Neto SBC (2013). Aspectos psicológicos de mães de crianças em tratamento
oncológico. Rev Saúde Desenvol Humano..

[5] Bona K, Dussel V, Orellana L, Kang T, Geyer R, Feudtner C, Wolfe J (2013). Economic Impact of Advanced Pediatric Cancer on
Families. J Pain Symptom Manage..

[6] Kohlsdorf M, Costa AL Junior (2011). Cuidadores de crianças com leucemia: exigências do
tratamento e aprendizagem de novos comportamentos. Estudos Psicol. (Natal)..

[7] Tsimicalis A, Stevens B, Ungar WJ, McKeever P, Greenberg M (2011). The Cost of Childhood Cancer from the Family's
Perspective: A Critical Review. Pediatr Blood Cancer..

[8] Hovén E, Essen LV, Norberg AL (2013). A longitudinal assessment of work situation, sick leave,
and household income of mothers and fathers of children with cancer in
Sweden. Acta Oncol..

[9] Eiser C, Upton P (2006). Costs of caring for a child with cancer: a questionnaire
survey. Child: Care, Health Develop..

[10] Deon KC, Santos DMSS, Reis RA, Fegadolli C, Bullinger M, Santos CB (2011). Tradução e adaptação cultural para o Brasil do DISABKIDS
- Atopic Dermatitis Module. Rev Esc Enferm USP..

[11] Guillemin F, Bombardier C, Beaton D (1993). Crosscultural adaptation of health-related quality of
life measures: literature review and proposed guidelines. J Clin Epidemiol..

[12] Herdman M, Fox-Rushby J, Badia X (1998). A model of equivalence in the cultural adaptation of
HRQol instruments: the universalist approach. Qual Life Res..

[13] Hasselmann MH, Reichenheim ME (2003). Adaptação transcultural da versão em português da
Conflict Tactics Scales Form R (CTS-1), usada para aferir violência no casal:
equivalência semântica e de mensuração. Cad Saúde Pública..

[14] Moraes CL, Hasselmann MH, Reichenheim ME (2002). Adaptação transcultural para o português do instrumento
Revised Conflict Tactics Scales (CTS2), utilizado para identificar violência entre
casais. Cad Saúde Pública..

[15] Pasquali, L (1999). Instrumentos psicológicos: Manual Prático de
Elaboração.

[16] Instituto Brasileiro de Geografia e Estatística(IBGE) (2000). Tabela 1.2.1 - Populaçăo residente, por cor ou raça, segundo a
situação do domicílio e os grupos de idade - Brasil - 2000.

[17] (1996). Lei n.9.394 de 20 de dezembro de 1996. Estabelece as diretrizes
e bases da educação nacional.

